# Extramedullary plasmacytoma of the orbit with intracranial invasion: A case report

**DOI:** 10.1097/MD.0000000000033920

**Published:** 2023-06-16

**Authors:** Yoo Jin Kim, Sang Woong Moon, , In-Ki Park, Jae-Ho Shin

**Affiliations:** a Division of Ophthalmology, Department of Medicine, Graduate School, Kyung Hee University, Seoul, Korea; b Department of Ophthalmology, Kyung Hee University Hospital at Gangdong, Kyung Hee University School of Medicine, Seoul, Korea; c Department of Ophthalmology, Kyung Hee University Hospital, Kyung Hee University School of Medicine, Seoul, Korea.

**Keywords:** extramedullary plasmacytoma, intracranial invasion, orbit, orbital plasmacytoma

## Abstract

Plasmacytoma is a disease caused by the proliferation of monoclonal immunoglobulin-producing plasma cells and divided into multiple myeloma, solitary bone plasmacytoma, and extramedullary plasmacytoma. We report a case of orbital extramedullary plasmacytoma invading the dura mater in a patient with exophthalmos and diplopia.

**Patient concerns:**

A 35-year-old female patient with exophthalmos in the right eye and diplopia visited the clinic.

**Diagnoses:**

The thyroid function tests showed nonspecific results. Orbital computed tomography and magnetic resonance imaging revealed a homogeneously enhancing orbital mass infiltrating the right maxillary sinus and adjacent brain tissue in the middle cranial fossa through the superior orbital fissure.

**Interventions:**

To diagnose and alleviate the symptoms, an excisional biopsy was performed, which revealed a plasmacytoma.

**Outcomes:**

One month after the surgery, the protruding symptoms and eye movement restriction in the right eye improved, and the visual acuity in the right eye was restored.

**Lessons:**

In this case report, we present a case of an extramedullary plasmacytoma that originated in the inferior wall of the orbit and invaded the cranial cavity. To our knowledge, no previous reports have described a solitary plasmacytoma that originated in the orbit, causing exophthalmos and invading the cranial cavity at the same time.

## 1. Introduction

Plasma cell dyscrasia is a rare condition in which mature plasma cells proliferate and produce monoclonal immunoglobulins. It varies from monoclonal gammopathy to multiple myeloma. Plasmacytomas are divided clinically and pathologically into 3 categories: multiple myeloma, solitary bone plasmacytoma, and extramedullary plasmacytoma. Of these, multiple myeloma originating from the bone marrow is the most commonly occurring disease.^[1,2]^ Conversely, bone-derived solitary bone plasmacytoma and soft tissue-originated extramedullary plasmacytoma rarely occur (<5% of cases). Extramedullary plasmacytoma usually manifests in the upper respiratory, head, and neck regions; therefore, a solitary orbital plasmacytoma is extremely rare.^[3–5]^

Solitary extramedullary plasmacytomas of the eyeball, orbit, and periorbital region reported in Korea include 2 cases of upper eyelid, 1 case of lacrimal drainage system, 2 cases of lacrimal gland, 1 case of anterior uvea, and 1 case confined to the orbit.^[6–12]^ One case of solitary bone plasmacytoma with primary bone destruction and invasion into the cranial cavity has been reported; however, an extramedullary plasmacytoma with an orbital origin and invasion of intracranial brain parenchyma has not yet been reported.^[13]^ Here, we report a case of extramedullary plasmacytoma that originated in the orbit, invading the middle cranial fossa and brain parenchyma by passing through the superior orbital fissure.

Informed written consent was obtained from the patient for publication of this case report and accompanying images.

No ethical approval was obtained, because this study was a retrospective case report and did not involve a prospective evaluation.

## 2. Case report

**Figure 1.**
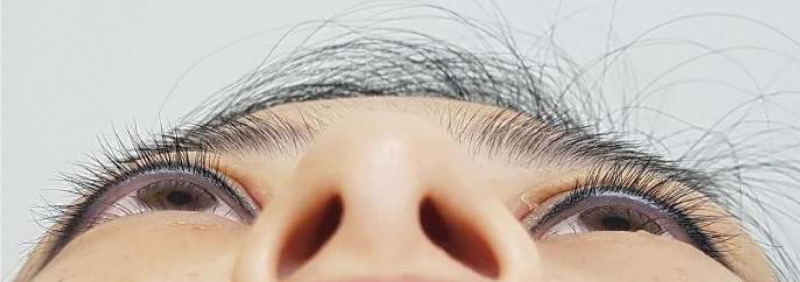
Exophthalmos of the right eye. Using the Hertel exophthalmometer, the right eye was 6 mm more protruded than the left eye.

A 35-year-old female patient visited our clinic complaining of protrusion of the right eye, which had occurred 1 month earlier. The patient had no specific medical or surgical history. Her visual acuity was 0.5 and 0.9 in the right and left eyes, respectively. The intraocular pressure was 16 and 18 mm Hg in the right and left eyes, respectively. In the Hertel exophthalmometer test, the right eye was 6 mm more protruded than the left eye (Fig. 1), exotropia of 10 prism diopters was measured at both near and far distances in the strabismus test, and she complained of diplopia in the full field. In the anterior segment examination, the eyelid edema and mass were not palpable, and there were no ophthalmologic findings other than exophthalmos.

**Figure 2.**
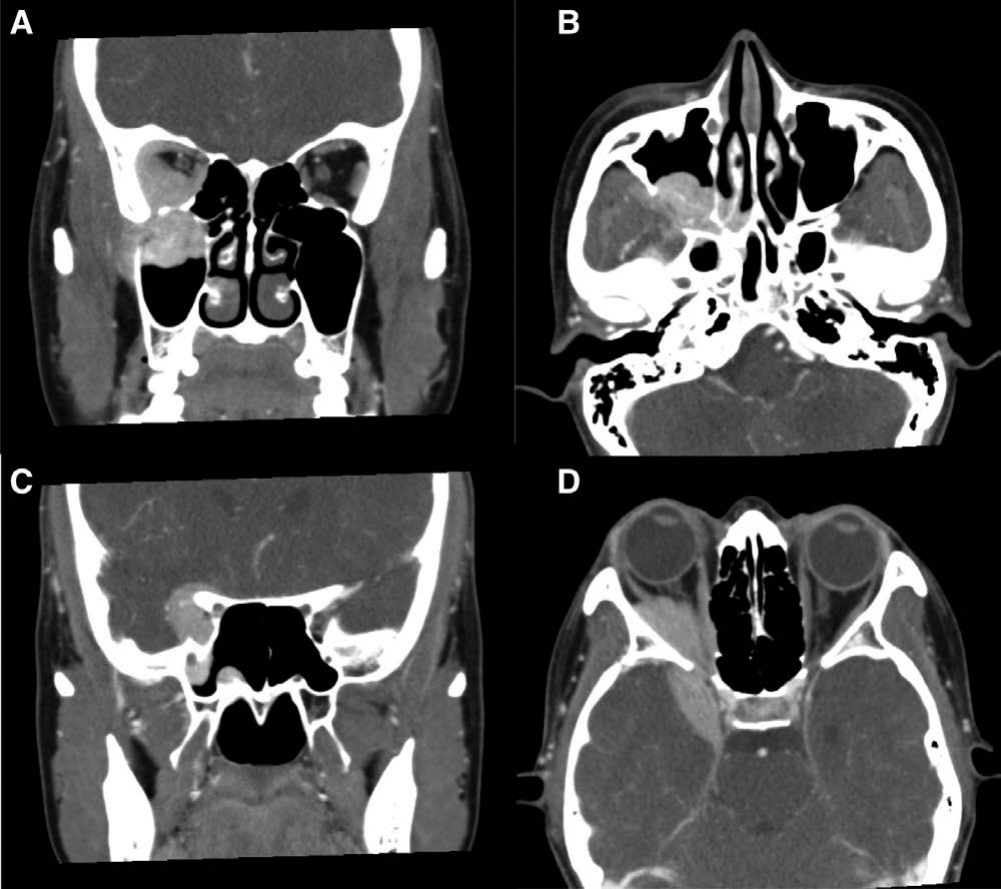
Orbital computed tomography scans show a well-enhancing tumor. (A) Axial and (B) coronal views of the orbital mass in the retrobulbar area extending into the right maxillary sinus. (C) Axial and (D) coronal views of the same mass extending into the right middle cranial fossa through the superior orbital fissure and invading the adjacent brain tissue.

**Figure 3.**
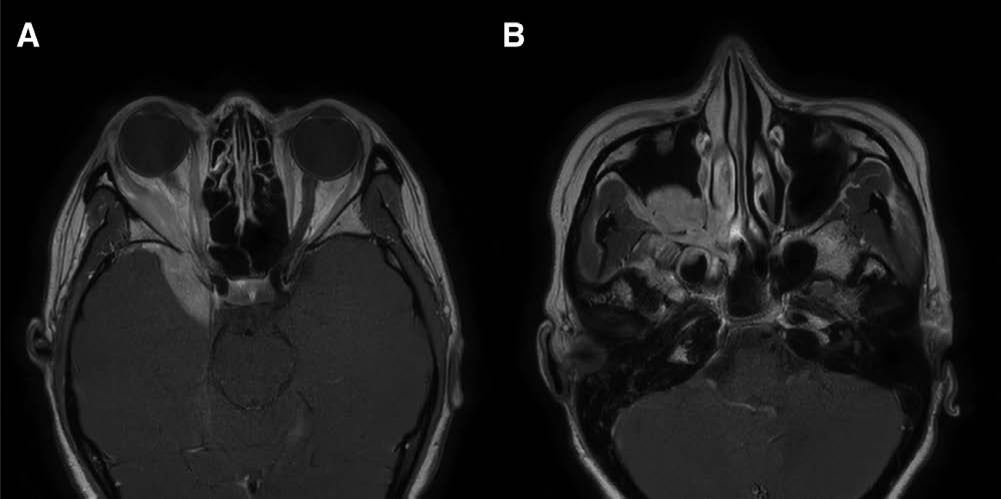
Axial scans of T1-weighted multipoint Dixon sequence in magnetic resonance imaging of the tumor. The tumor shows high signal intensity in, (A) the right retrobulbar area and the middle cranial fossa and (B) the right maxillary sinus.

On orbital computed tomography and magnetic resonance imaging tests, a uniformly contrasted mass measuring 4.4 × 3.0 × 3.0 cm was found over the right maxillary sinus and posterior region of the eye. As the mass passed through the superior orbital fissure and extended to the middle cranial fossa, invasion of the cranial cavity and brain parenchyma was observed (Figs. 2 and 3).

**Figure 4.**
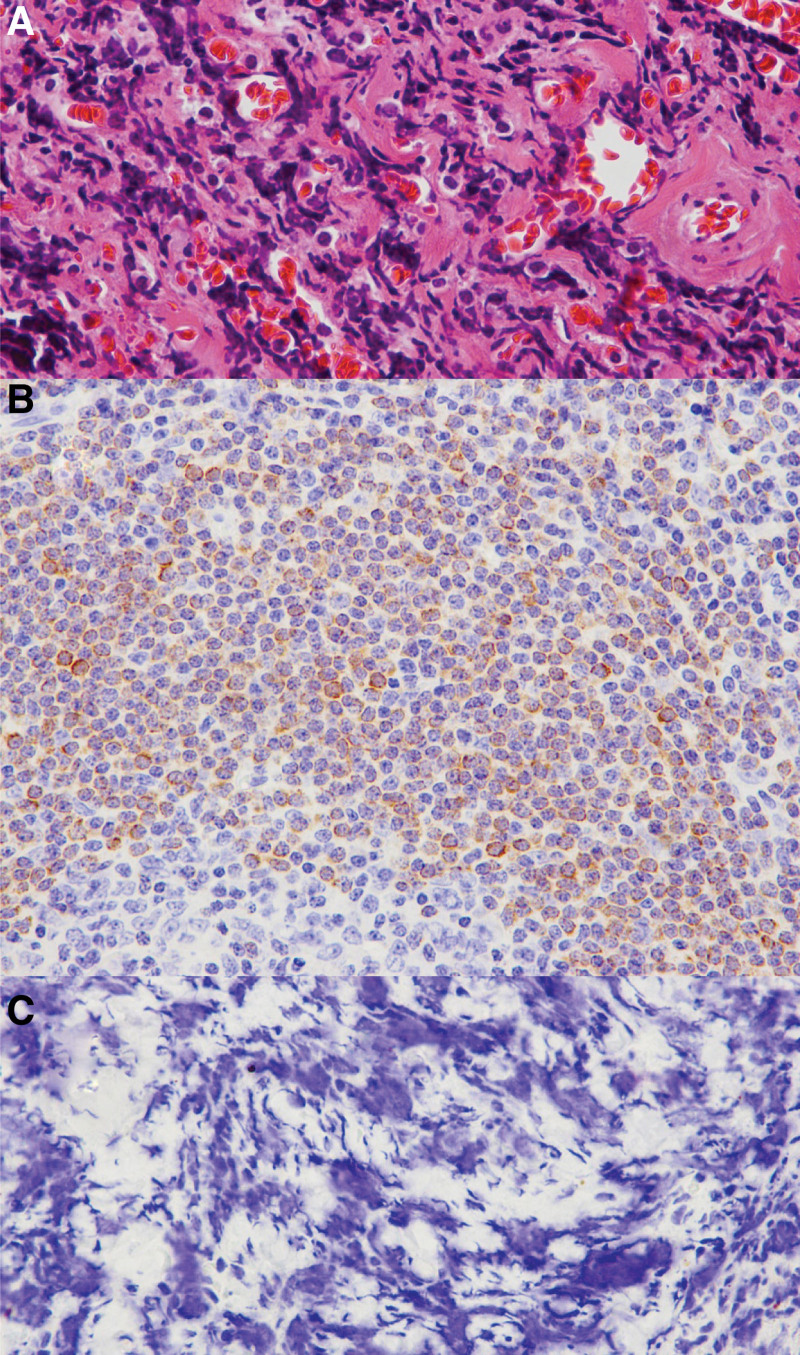
Histopathologic findings of the mass. (A) Monotonous plasma cells with an eccentric nucleus and basophilic cytoplasm (H&E, ×400). Negative staining of tumor cells for, (B) CD3, and (C) CD20 (×400). H&E = hematoxylin and eosin.

For diagnosis, tumor resection and biopsy were performed under general anesthesia. After incising the conjunctiva and lower eyelid retractor along the lower part of the lower eyelid plate, the orbital septum and posterior septal adipose tissue were removed, and the inferior orbital wall was exposed. The orbital mass was removed as much as possible, and a histopathological examination was performed. In hematoxylin and eosin immunohistochemistry, plasma cells with eccentric nuclei, basophilic cytoplasm, and perinuclear zona pellucida were characteristically observed. CD3 and CD20 negative findings, along with lambda light chain positive and kappa chain negative findings, were obtained, and plasmacytoma was diagnosed (Fig. 4).

One month after the surgery, the protruding symptoms and eye movement restriction in the right eye improved, and the visual acuity in the right eye was restored to 0.9. No special complications were observed after surgery, and the patient was transferred to another hospital for an additional evaluation at the Hematology and Oncology Department for plasmacytoma, and no follow-up examination was performed. He continued the chemotherapy and radiation therapy for the treatment of lung cancer and brain metastatic cancer. Despite treatment, the symptoms worsen and the patient was deceased after 4 months.

## 3. Discussion

Plasmacytoma is a disease caused by the indiscriminate proliferation of monoclonal immunoglobulin-producing plasma cells and presents various clinical and pathological aspects. They are divided into multiple myeloma, solitary bone plasmacytoma, and extramedullary plasmacytoma (EMP). Of these, multiple myeloma occurs most frequently, and solitary bone plasmacytoma and extramedullary plasmacytoma occur rarely, with incidences of 5% and 2%, respectively. ^[1,2]^ Approximately 90% of extramedullary plasmacytomas occur predominantly in the head and neck, particularly in the walls of the upper respiratory tract, including the nasal cavity, paranasal sinuses, oropharynx, salivary glands, and larynx, and orbital primary cases are extremely rare.^[1]^

Extramedullary plasmacytoma presents with various symptoms, depending on the area within the eyeball and orbit. The following symptoms have been reported in cases in Korea: a palpable painless mass, reduced visual acuity, exophthalmos, blepharoptosis, and lacrimation owing to masses that invaded regions involving the upper eyelid, lacrimal drainage system, lacrimal gland, anterior uvea, and orbit. No previous reports have described a solitary plasmacytoma that originated in the orbit, causing exophthalmos and invading the cranial cavity at the same time.

The diagnosis of an EMP requires confirmation of plasma cell proliferation and antibody production on histopathological examination; however, it is difficult to differentiate it from multiple myeloma. Systemic examination is required to differentiate it from multiple myeloma,4 which appears as a systemic disease. Monoclonal antibody titers should be normal in serum and urine protein electrophoresis tests, and no specific findings should be observed in computed tomography, bone marrow aspiration, positron emission tomography, or general bone imaging tests. EMP can be diagnosed when there are no findings, such as hypercalcemia, renal failure, anemia, and lytic bone change, which are characteristics of multiple myeloma.^[1,5]^

Thuro et al ^[14]^ reported that orbital plasmacytomas can be anatomically divided into 4 types. Plasmacytomas invaded the superior temporal orbit, epidural space, and temporal fossa in 15 of 30 patients (50%) and were confined to the orbit in 7 patients (23%). To a lesser extent, primary tumors in the nasal cavity were found in 4 patients (13%), and tumors that originated in the orbital floor and invaded the soft tissue of the skin were confirmed in 3 patients (13%).

As plasmacytoma is sensitive to radiation, the main treatment method is radiation therapy, and an irradiation of 40 to 45 Gy or more to the lesion produces the best results for local control without any side effects. A surgical excision is also possible if the lesion is located in an easily accessible location, and it is known that the recurrence rate is low when combined with radiation therapy. Although there are reports of studies on chemotherapy, it is mainly performed when plasmacytoma has progressed.^[2]^

Extramedullary plasmacytoma has a relatively good prognosis, progressing to multiple myeloma in 10% to 35% of cases, whereas isolated osteoplasmacytoma progresses to multiple myeloma in 30% to 75% of cases.

In our case, the protruding symptoms and right eye movement restriction of the patient improved within one month after the surgery, and the visual acuity in the right eye was restored to 0.9. No special complications were observed after surgery, and the patient was transferred to another hospital for an additional evaluation at the Hematology and Oncology Department for plasmacytoma, and no follow-up examination was performed.

Although further follow-up could not be performed as the patient in this case was transferred to another hospital, it is important to rule out multiple myeloma by performing systemic examinations, such as serum and urine protein electrophoresis, bone marrow aspiration, and whole-body imaging. In addition, intraorbital masses and other invasion sites should be identified through specific imaging of the orbit, and surgical resection and radiation treatment should be performed.

## 4. Conclusion

In this case report, we present a case of an extramedullary plasmacytoma that originated in the inferior wall of the orbit and invaded the cranial cavity, but not the temporal region, which is usually found in the orbit, in Korea, based on a literature review. In addition, liver plasmacytoma should be considered.

## Author contributions

Conceptualization: Yoo Jin Kim, Sang Woong Moon.

Data curation: Yoo Jin Kim, Sang Woong Moon.

Projection administration: Yoo Jin Kim, Sang Woong Moon, In-Ki Park, Jae-Ho Shin.

Supervision: Jae-Ho Shin.

Visualization: Yoo Jin Kim, Sang Woong Moon.

Writing – original draft: Yoo Jin Kim, Sang Woong Moon.

Writing – review & editing: In-Ki Park, Jae-Ho Shin.
